# Outcome after polyaxial locking plate osteosynthesis in proximal tibia fractures: a prospective clinical trial

**DOI:** 10.1186/s12891-021-04158-z

**Published:** 2021-03-18

**Authors:** Dominik Völk, Markus Neumaier, Heike Einhellig, Peter Biberthaler, Marc Hanschen

**Affiliations:** 1 Department of Trauma Surgery, Technical University of Munich, School of Medicine, Klinikum rechts der Isar, Ismaninger Strasse 22, 81675 Munich, Germany; 2 Department of Trauma and Orthopaedic Surgery, Klinikum Freising, Freising, Germany; 3 Department of Radiology, Technical University of Munich, School of Medicine, Klinikum rechts der Isar, Munich, Germany

**Keywords:** Proximal tibia fracture, Clinical trial, Polyaxial locking plate, Outcome, Osteosynthesis

## Abstract

**Background:**

The aim of this study was to evaluate the clinical and/or radiologic outcome using different polyaxial locking plates for the treatment of proximal tibia fractures, the Non-Contact-Briding plate (NCB-PT®) by Zimmer or the Variable Angle Locking Compression Plate (VA-LCP®) by Synthes.

**Methods:**

This study enrolled 28 patients with proximal tibia fractures (AO/ OTA 41 B-C) and indication for locking plate osteosynthesis. All patients were treated with a polyaxial locking plate system. Depending on the fracture morphology, patients were either treated with a NCB-PT® or VA-LCP®. The implant was chosen according to the surgeon’s experience and preference, in case of a higher degree of comminution the tendency was observed to use the NCB-PT® plate. After a time interval of 12 months postoperative we conducted clinical (e.g. range of motion, the Rasmussen score) and radiological (e.g. primary/secondary loss of reduction) follow-ups.

**Results:**

Patients provided with the NCB-PT® (9 patients) showed longer operation time, use of longer implants, longer interval from injury to surgery and lower clinical scores after the 12 months follow-up compared with the VA-LCP® group (19 patients). Interestingly, the results showed no significant differences regarding the clinical and radiologic outcome.

**Conclusions:**

The small number of patients as well as the heterogeneity of fractures constitute a limitation of this study. Nevertheless, the differentiated use of implants is associated with comparable clinical and radiological outcomes. This trial emphasizes the need for further prospective randomised trials with higher patient numbers.

**Trial registration:**

Retrospectively registered 21.12.2020. Registration number NCT04680247.

## Background

Proximal tibia fractures constitute a small fraction (1.2%) of adult fractures [1]. Aetiologically, there are two main injury mechanisms: high energy trauma (e.g. traffic accidents), which appears mainly in younger patients, and low energy trauma, which frequently appears in older patients commonly in connection with reduced bone density [2]. The main therapeutic goals in the treatment of fractures of the proximal tibia are the reconstruction of the joint surface, the mechanical axis, the length and the rotation as well as addressing concomitant injuries [3].

The standard procedure in the treatment of articular fractures of the proximal tibia remains to be the surgical treatment. The open reduction and internal fixation (ORIF) with the use of an anatomical preshaped locking plate has been established as the standard procedure [4–6]. Regarding the primary and secondary outcome (soft tissue damage, loss of reduction, malalignment), this technique has shown excellent results in the current literature [6–9].

Nevertheless, angular stable locking plates of the first generation had some disadvantages. Due to their anatomical preshaped design and the integrated thread in the plate the positioning of screws in the bone is limited. In conjunction with the predetermined position of the screw there is a risk of primary screw misplacement or rather fixation of the screw in a section with low bone quality. Secondary loss of reduction and/or screw loosening could be the consequences [10].

These disadvantages lead to further development, which resulted in polyaxial locking plate systems. The idea behind these locking plates was to allow the surgeon a deviation of the screw axis, without losing the advantage of the mechanical bridging of the fracture by the locking mechanism between plate and screw. This deviation of the screws gives the surgeon the opportunity for a more precise positioning of the screws when faced with regional differences in bone quality [11] or multifragmentary fracture patterns.

Currently, different polyaxial implants from different manufacturers are available for the treatment of proximal tibia fractures. These various polyaxial implants have a variety of different characteristics, e.g. the use of different materials, different thickness of the plate, and different diameters of the screws. Furthermore, the design of the screw heads and plate holes, constituting the locking mechanism, vary significantly.

Regarding the numerous differences between the polyaxial locking systems the question is raised, whether these differences have an impact on the outcome when used for treatment of tibial head fractures.

The development of monoxial locking plates constituted a milestone for the treatment of proximal tibia fractures [6–9]. The evolution of this technique, leading to polyaxial locking systems, has the potential to further improve this treatment. First clinical studies comparing monoaxial and polyaxial systems could find advantages in favour of the polyaxial systems [10]. Nevertheless, clinical studies comparing the outcome of different polyaxial locking plates are still missing. Therefore, this prospective clinical trial analysis the outcome of patients treated with two different polyaxial locking plates, the NCB-PT® by Zimmer (Winterthur, Switzerland) and the VA-LCP® by Synthes (West Chester, Pennsylvania, USA), used for the treatment of complex proximal tibia fractures.

## Methods

### Patients

**Fig. 1:**
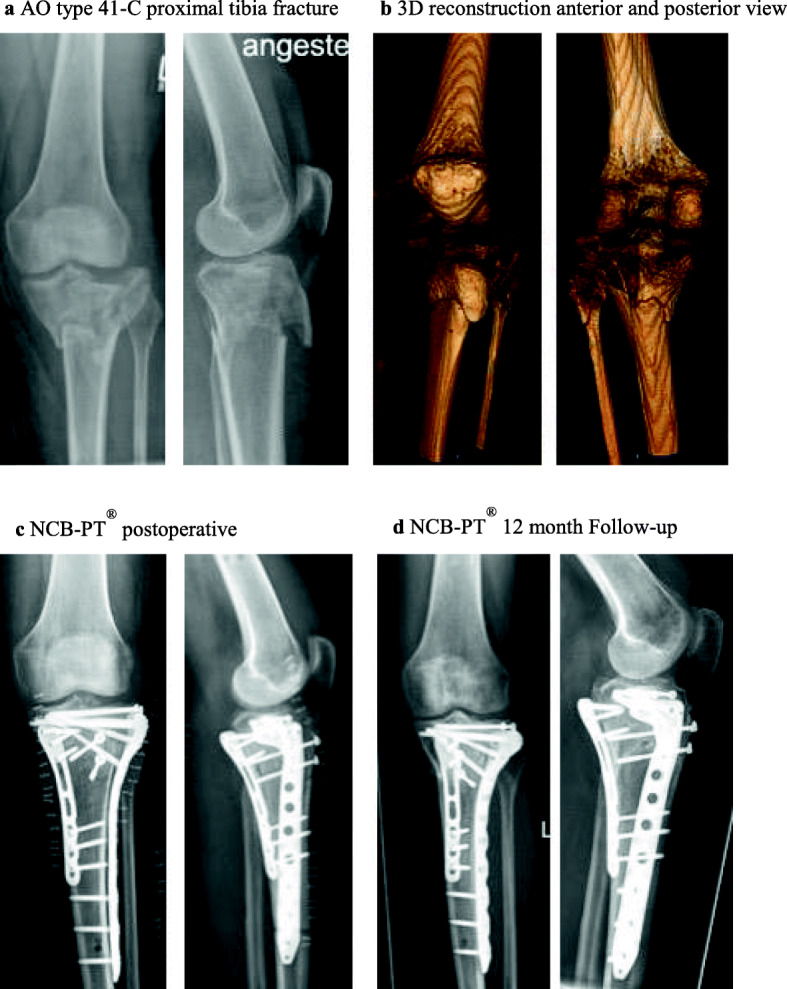
This figure shows an exemplary case of a 64-year old patient treated with a NCB-PT® system. The patient suffered from a AO type 41-C proximal tibia fracture (**a-b**) after a skiing accident. Initial fixation with an external fixator allowed the soft tissue to consolidate. Due to soft tissue concerns, a staged procedure was chosen for definitive reconstruction. In the first step we conducted a plate osteosynthesis after reduction from posteromedial with a Synthes LCP and the patient in prone position. For the second step we performed a standard anterolateral approach. After a horizontal arthrotomy and visualisation of the entire lateral plateau we elevated the central indented fragment and supported it with an allogeneic cancellous bone graft. After reaching a satisfactory reduction of the lateral compartment we inserted a 7-hole NCB-PT® plate as well as two proximal 4.0 mm lag screws. In a final step we performed a refixation of the tibial tuberosity with two 4.0 mm lag screws The presented images show the postoperative radiologic controls after the final surgery (**c**) and the image of our radiologic follow-up one year after surgery for NCB-PT® patients (**d**)

**Fig. 2:**
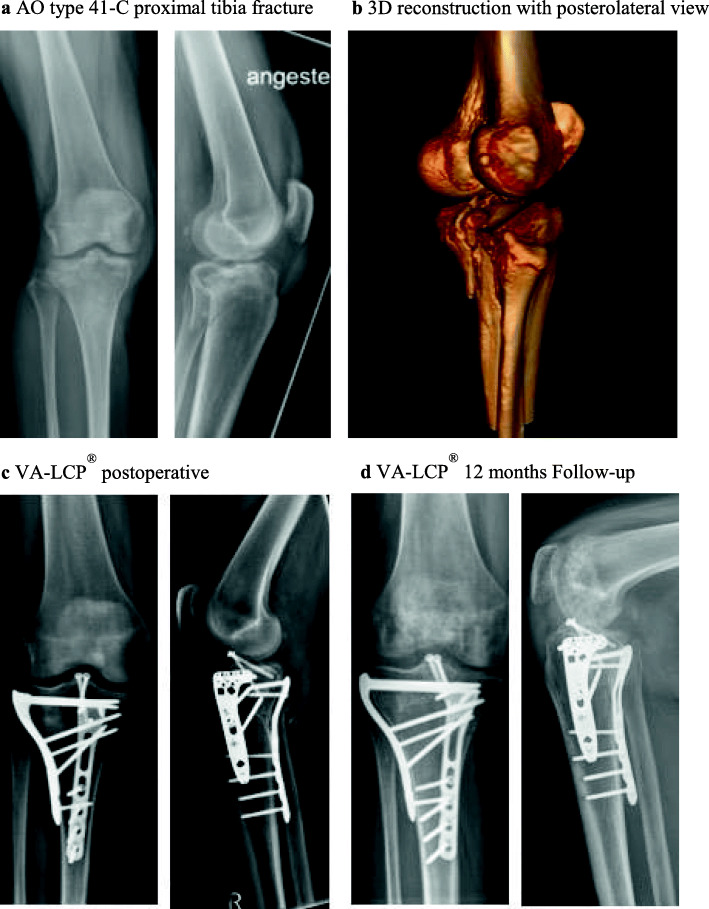
The second presented case is a 60-year old patient with an AO type 41-C proximal tibia fracture (**a-b**) after a fall from under 2 m. Due to soft tissue concerns, a staged procedure was chosen for definitive reconstruction. First, we performed a posteromedial approach. After reduction and temporary K-wire fixation we inserted a Synthes LCP plate for posteromedial osteosynthesis. In the same surgery, we conducted a standard anterolateral approach and horizontal arthrotomy. The main posterolateral fragment was reduced until we reached a stepless result. After insertion of an allogeneic cancellous bone graft, we preformed the fragment fixation with a 4-hole VA-LCP®. Following further consolidation of the soft tissue, we conducted an arthroscopy of the knee and screw osteosynthesis of the eminentia intercondylaris. The images show the postoperative radiologic controls after the final surgery (**c**) and the image of our radiologic follow-up one year after surgery for VA-LCP® patients (**d**)

Prior to the onset of the study, the approval by the medical ethics committee of the Technical University of Munich (TUM) (Trial Number: 5923/13) was obtained. During a period from October 2013 to December 2015 we enrolled 28 patients aged 25 to 82 into our study. All patients suffered a fracture of the proximal tibia. The fractures were classified according to the AO/OTA classification [12]. Every one of the 28 patients had an indication for locking plate osteosynthesis. We included all type 41-B [13] fractures and all type 41-C [12] fractures. Pathological fractures, pregnancy, adolescence (age < 18 y), prisoners and patients currently put under tutelage were excluded. The patients were scheduled for a locking plate osteosynthesis with either the NCB-PT® system (Fig.1) or the VA-LCP® system (Fig.2). The implant was chosen according to the surgeon’s experience and preference, in case of a higher degree of comminution a tendency towards the use of the NCB-PT® plate was observed. Besides the degree of comminution, age, bone quality, allergies, and distal fracture extension were among the criteria taken into account to determine the implant type.

### Implants

The NCB-PT® is an anatomical preshaped polyaxial locking plate. In addition to its preshaped design, the head of the plate has a posterior tilt of 6° matching the physiological slope of the tibial plateau. The plate consists of a titanium alloy (TiAIV) and is available with 2 or 3 proximal holes. The NCB-PT® system features cancellous screws with a 5.0 mm and cortical screws with a 4.0 mm diameter. The implant can be inserted in MIS (minimally invasive surgical) technique using a radiolucent target device.

The polyaxial design allows a variation of the screw position up to 30°. Conventional screws are inserted and later locked with a specially designed locking cap. Thereby it is possible to use the conventional screw as a lag screw followed by locking the same screw. Another advantage of the secondary locking mechanism is the sustained “feeling for bone quality” while tightening the screws [14]. Unfavourable is the large profile height of the implant.

The VA-LCP® is also an anatomical preshaped locking plate. This plate is made out of steal and is available in two versions, which either have a large or a small bend. Both versions are assembled with 3.5 mm cortical and cancellous screws. Using the radiolucent target device, screws in the diaphyseal region can be inserted in MIS technique. Specially shaped holes in the plate and a specially designed thread consisting of four columns, allow a variation of the screw position up to 30° around the central axis of the plate hole. So called combined holes enable the use of locking screws or lag screws at the same plate position. The combined holes can only be found at the shaft part of the plate therefore it is not possible to insert lag screws through the proximal part of the plate. In contrast to the NCB-PT®, the VA-LCP® is a so called “low-profile plate” with a low profile height.

### Surgical technique

The surgical technique was standardized, as far as possible. The anterolateral approach was used in all cases. Depending on the fracture type and the damaged column the standard approach was supplemented with either a posterolateral approach [15] or a posteromedial approach [16]. After arthrotomy and suturing of the lateral meniscus the fracture was reduced. This was performed under direct visual control of the joint surface and/or image intensifier. K-wires and/or a reduction forceps was used to secure the reduction. Bone defects after reduction were filled with either an autologous cancellous bone graft or an injectable bio-ceramic composite (Cerament®, Bonesupport, Sweden).

Afterwards the locking plate was inserted and temporarily fixed to the bone with K-wires. After checking the correct position of the plate with an image intensifier the screws were applied. In case of VA-LCP®, additional lag screws were inserted at discretion of the surgeon. All patients received a perioperative single shot antibiotic. Our postoperative procedure consisted of a partial weight bearing for 6 weeks for all patients. Regarding the allowed range of motion we distinguished two different groups: patient who received an arthrotomy and a refixation of the meniscus ought to comply with a limited range of motion for 6 weeks overall (week 1–2 30/0/0, week 3–4 60/0/0, week 5–6 90/0/0, week 7 free RoM). Patients with no arthrotomy were allowed free range of motion immediately after surgery.

### Intraoperative data

To collect the intraoperative data, we analysed the intraoperative protocols of each patient to assess the operative time, length of the implant and the use of a bone graft or bone void filler. In addition, we used surgical protocols and patient reports to gather data about intraoperative arthroscopy, use of a reduction aids and use of lag screws.

### Postoperative clinical follow-up

We conducted the follow up after 12 months. The clinical evaluation was performed in our trauma outpatient clinic. With the help of standardized questionnaires, we examined amongst others: the range of motion, cruciate ligament/ collateral ligament instability and meniscus signs to measure the clinical outcome. We also collected data from 5 different knee scoring systems, the Tegner score [13], the Rasmussen score (clinical part) [17], the Oxford knee score [18] the Munich knee questionnaire [19] and the Lysholm score [13]. To measure the patient satisfaction we used the SF36 (36-Item Short-Form Health Survey) [20].

The postoperative clinical data, obtained via clinical examination and scoring systems at our 12-months follow-up, was compared against self-assessment of the patients’ pre-operative status. Data was achieved by sending out self-assessment sheets to the patients at 3–6 month following surgery, kindly asking them for self-assessment of the preoperative status using self-assessment surveys.

### Postoperative radiological follow-up

Besides the clinical data the primary outcome measurements also included standardized, blinded radiological evaluation. We conducted X-ray examinations in two plains (AP, lateral view) on the second postoperative day as well as 12 months following surgery and examined them for signs of screw misplacement, primary/secondary loss of reduction, non-union and malalignment. Furthermore we collected the data of the radiological part of the Rasmussen score which contains articular surface depression, condylar widening and fragment angulation [17].

### Statistics

The statistical analysis was performed with the program GraphPad Prism 6 (GraphPad Software Inc., La Jolla, CA, USA). To check the data for standard distribution we used the D’Agostino omnibus K2 test. For continuous parametric variables we used the Student’s t-test, for non-parametric variables the Mann-Whitney U test and for binominal variables the Fisher’s exact test. In all analysis the significance level was set at a *p*-value < 0,05, we plotted the data as mean values ± SEM.

## Results

### Epidemiological data

We couldn’t find any statistically significant differences in patient characteristics. The mean age was 57.1 years for the VA-LCP® group and 53.6 years for the NCB-PT® group. As a result of the surgeon’s assessment of the injury and implant choice, there was an imbalance noticeable regarding the injury severity within the groups. The percentage of type-C fractures was lower in the VA-LCP® group (26%) as compared with the NCB-PT® group (78%). Regarding the trauma specifics, road traffic accidents and sports accidents represent the major cause (Table 1).

**Table 1:** Patient and injury data

Characteristics	NCB-PT® (*n* = 9)	VA-LCP® (n = 19)
sex (m:f)	6:3	9:10
age (years)	53.6	57.1
trauma mechanism		
traffic accident	3	7
sport accident	4	4
fall < 2 m hight	1	5
other	1	3
AO classification		
41-A	0	0
41-B	2 (22%)	14 (74%)
41-C	7 (78%)	5 (26%)
fractured side (l:r)	5:4	11:8

28 patients which sustained a proximal tibia fracture (AO type 41-B/C) were included in our study. The patients were randomized into two groups and either received a VA-LCP® (Variable Angle Locking Compression Plate) system or a NCB-PT® (Non-Contact Bridging for Proximal Tibia) system for fracture treatment. Regarding the patient and injury data we could not find any statistically significant differences

### Intraoperative data

**Fig. 3:**
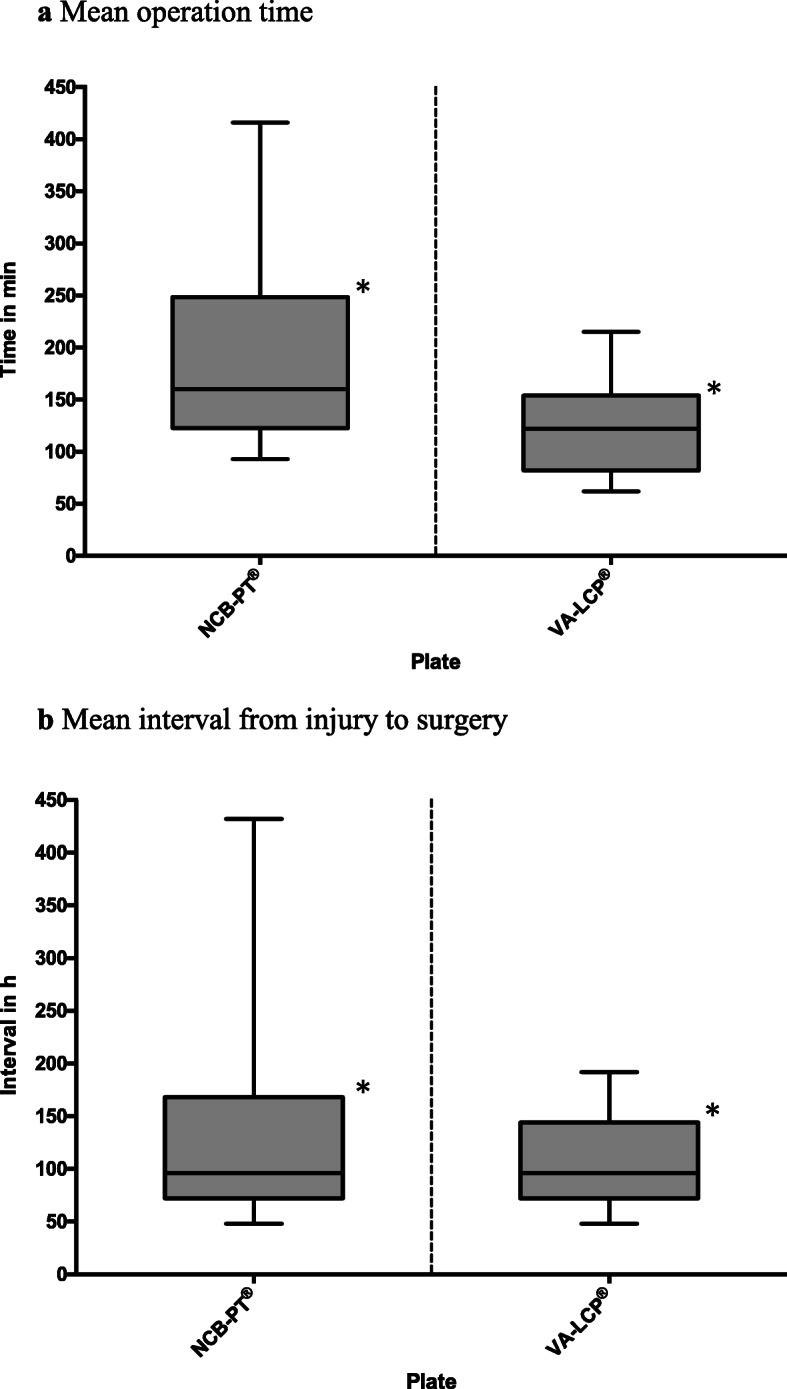
Analysing the intraoperative data between the patients treated with a VA-LCP® or NCB-PT® we could determine a significant difference regarding the mean operation time (panel **a**) and the interval from injury to surgery (panel **b**). As a result of the heterogeneity of fracture types both proofed to be statistically significant (operation time, *p*-value 0.0335; injury to surgery interval, p-value 0.0004)

Intraoperative data showed a statistically significant longer surgery duration (NCB® 196.3 min vs. VA-LCP® 121.7 min) (Fig. 3) and use of larger implants (NCB® 8.1 holes vs. VA-LCP® 5.1 holes) within the NCB-PT® patient group (*p-value: 0.0335 and 0.0007*) (Table 2). In addition to a longer operative time the NCB-PT® patient group also had a considerably longer time from the point of injury to the surgical treatment (NCB® 141.3 h vs. VA-LCP® 103.6 h, *p-value 0.0004*) (Fig.3) due to the higher number of severe type-c fractures in this group. Other intraoperative details such as use of bone void fillers or intraoperative arthroscopy were comparable between the groups or had a small non-statistically significant difference (Table 2).

**Table 2:** Intraoperative data

Characteristics	NCB-PT® (*n* = 9)	VA-LCP® (*n* = 19)
Duration trauma/ surgery (h)	141.3 (5.9 d)	103.6 (4.3 d)
Surgery duration (min)	196.3	121.7
Intraoperative arthroscopy	1	2
Bone void filler		
Injectable bio-ceramic composite (Cerament® BVF)	0	2
Cancellous bone graft	1	3
Distal extended fracture	4	4
plate type		
7-hole NCB® /4-hole VA-LCP®	6 (66.7%)	13 (68.4%)
9-hole NCB® /6-hole VA-LCP®	2 (22.2%)	4 (21.2%)
13-hole NCB® /10-hole VA-LCP®	1 (11.1%)	2 (10.5%)

Using surgical protocols and reports of each patient we collected intraoperative data for each patient. Analysing this data and comparing the VA-LCP® group with the NCB-PT® group we found a considerable difference between both groups regarding the mean time from injury to surgery as well as the mean operation time. Both were statically significant. The other intraoperative parameters showed no significant difference

### Postoperative clinical follow-up

In the analysis of the primary clinical and additional secondary clinical data, no significant difference between both groups could be detected.

**Fig. 4:**
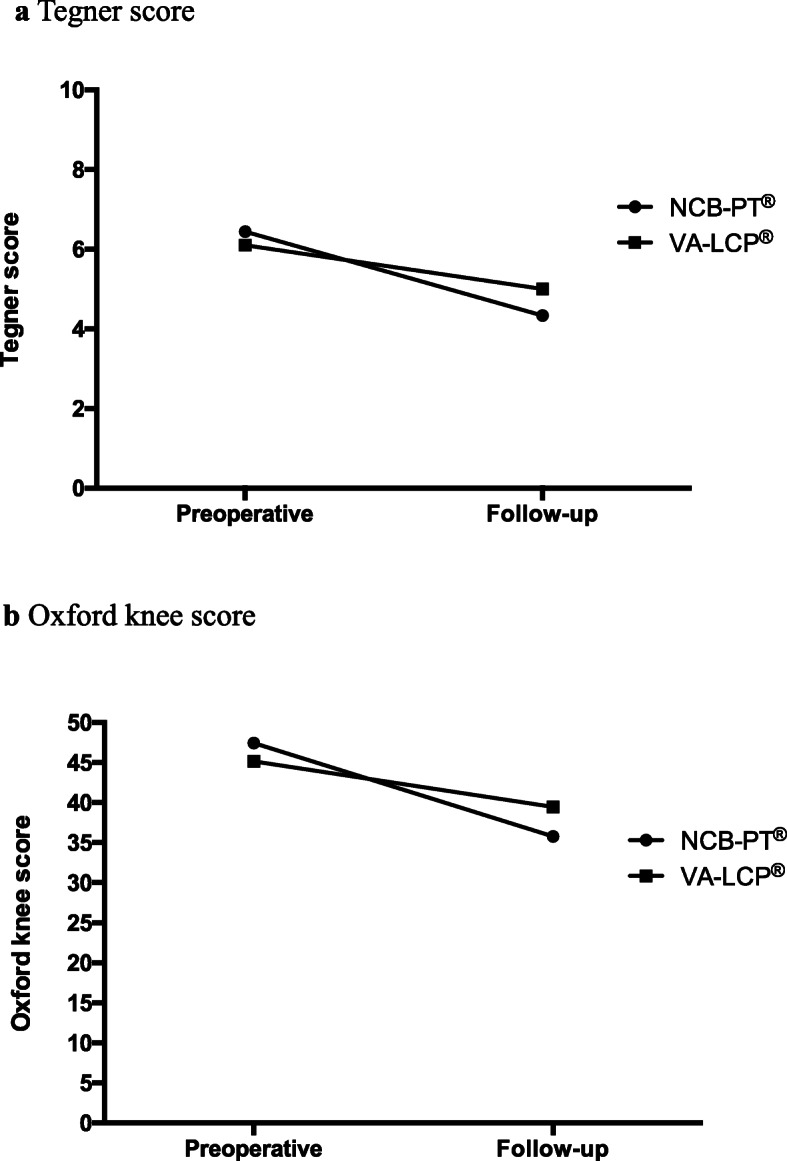
In order to reach a comprehensive analysis of the clinical outcome we used five different scoring systems. Among those were the Tegner score (panel **a**) and Oxford knee score (panel **b**). Each one of the scores was evaluated by patients’ self-assessment survey to reflect the situation before the accident and at our 12 months follow-up. In our analysis patients tended to reach comparable functional scores at the 12 month follow-up

In addition to the clinical parameters, we also used clinical scores further evaluate the outcome. We utilized the Lysholm score und Rasmussen score to reflect functionality and pain while the Tegner score depicts the sports activity and the Oxford knee score shows daily life activity and pain. The Munich knee questionnaire (MKQ) is a relatively new scoring system which includes pain, daily life and sports activity, as well as functionality In the statistical analysis of the scoring systems the Lysholm score (*p*-value: 0.03) and the MKQ (*p-value: < 0.0001*) showed statistically significant lower figures in the NCB-PT® patient group after the 12 months follow-up as compared to the VA-LCP® patients group (Table 3) as expected regarding the imbalance of severe type-c fractures between both groups. The remaining scoring systems and the sum scales of the SF 36 showed comparable results with no statistical significance (Table 3 and Fig.4). Complications were monitored in the patient population throughout the study course, 5 out of the 28 patients (18%) developed a complication during our 12 months follow up. Two of these patients required additional surgical procedure (Table 4).

**Table 3:** Clinical scores

Scoring system	Plating system	Preoperative (Self-assessment)	Follow-up (12 months)
Oxford knee score	NCB-PT® VA-LCP®	12.6 ± 0.4 14.8 ± 1.3	35.8 ± 3.5 39.5 ± 1.6
Tegner score	NCB-PT® VA-LCP®	6.4 ± 0.5 6.1 ± 0.4	4.3 ± 0.4 5.0 ± 0.8
Rasmussen score	NCB-PT® VA-LCP®	29.7 ± 0.2 28.8 ± 0.5	25.8 ± 1.4 26.4 ± 0.9
Lysholm score	NCB-PT® VA-LCP®	94.3 ± 4.5 91.2 ± 3.9	60.3 ± 9.5 81.0 ± 4.3
Munich knee questionnaire	NCB-PT® VA-LCP®	90.5 ± 0.01 74.7 ± 0.1	58.9 ± 0.1 63.5 ± 0.1
SF 36 physical health	NCB-PT® VA-LCP®	88.4 ± 2.2 76.7 ± 4.4	65.8 ± 7.1 65.9 ± 5.4
SF 36 mental health	NCB-PT® VA-LCP®	85.11 ± 2.9 75.9 ± 4.3	71.8 ± 5.8 72.8 ± 5.1

For the analysis of the clinical outcome we used five scoring systems as well as the 36-Item Short-Form Health Survey and evaluated their score before the accident and at our follow-up, 12 months after osteosynthesis of a proximal tibia fracture. Comparing the preoperative self-assessment scores and the follow-up scores of the NCB®-PT and VA-LCP® for each scoring system, the NCB® osteosynthesis showed lower functional scores in all follow-up scoring systems. Though only the results of the Lysholm score (*p*-value 0.024) and MKQ score (p-value < 0.0001) were statistically significant

**Table 4:** Complications

Patient	Complication	Revision	Second. bone void filler
34 y., m NCB-PT®	arthrofibrosis peroneal paralysis	no	no
61 y., m VA-LCP®	valgus deformity after compl bone healing	yes	yes (autologous cancellous bone graft)
73 y., f VA-LCP®	DVT (V. popl./fibul./tibial)	no	no
71 y., f VA-LCP®	DVT (V. popl./fem. Sup.)	no	no
59 y., m VA-LCP®	postop. Depression (dorsal articular surface)	yes	yes (Cerament®)

Throughout the 12 months follow-up the postoperative course of every patient was constantly monitored with radiological and clinical controls to detect any signs of a postoperative complication. Within our patient collective 5 postoperative complications appeared with two of them resulting in a operative revision, both out of the VA-LCP® group. A significant difference between the patients with a VA-LCP® or NCB-PT® system could not be identified

### Postoperative radiological follow-up

As well as the clinical Rasmussen score, the radiological part showed no difference postoperatively between both groups. The further parameters we evaluated did not show significant differences. The fracture healing proceeded satisfactory with no case of pseudarthrosis and only one case still in reparative phase up to the time of our follow-up. Regarding the alignment 3 patients from the NCB-PT® and 4 from the VA-LCP®-group had a malalignment.

## Discussion

The surgical reconstruction of complex proximal tibia fractures remains challenging, due to displacement, distinctive soft tissue damage and articular involvement [21, 22]. In addition the rising number of elderly patients with high comorbidity and poor bone quality further complicates the therapy [2]. ORIF treatment with preshaped locking plate systems showed excellent results concerning the therapy of these injuries [6–9]. The polyaxial locking plates represent a further development of this technique to address the described disadvantages especially the predetermined position of the screws. The NCB-PT® as well as the VA-LCP® allows a variation of screw positioning within specified limits. Besides these two products there are numerous other polyaxial plate systems for the proximal tibia available.

However, at the current moment there are no prospective studies including different polyaxial locking plates in proximal tibial fractures. This is the first clinical trial to analyse and compare the outcome of different polyaxial locking plate systems in this entity.

The previous studies mostly compare monoaxial and polyaxial locking plates except for the biomechanical study from Mehling and coworkers, which compares different polyaxial locking plates for distal radius fractures [23]. In a biomechanical study using synthetic bone and fixing a metaphyseal gap osteotomie with either a LISS® or NCB-PT® plate, both systems showed similar results regarding stiffness, strength to failure and resistance to plastic deformation [24]. Another biomechanical study also using synthetic bone compared the LCP® with the Polyax® (DePuy) and the NCB-PT® comes to the same conclusion saying there is no difference between the plates regarding physiological forces either at full or partial weight bearing [25]. We couldn’t find any biomechanical testing for the VA-LCP® used at the tibia. Only one study using this type of implant in olecranon fractures which showed significant better results in comparison to a monoaxial system [26]. In addition, the biomechanical studies, despite their imperative in studying these new implants, have a limited transferability on the clinical use. Of note, none of these studies simulate an osteosynthesis in osteoporotic bone. Especially in osteoporotic bone, the advantages of the polyaxial locking system could have a different impact on the stability of the osteosynthesis with either one of the implants due to the different characteristics of the plate systems (e.g. different screw diameters).

Jöckel and coworkers used the NCB-PT® in two prospective clinical trials in 36 and 86 patients with proximal tibia fractures. They reported a comparable outcome to the current literature after a follow up of 12 months [27, 28]. Neither biomechanical nor clinical studies could detect any differences when using polyaxial systems.

Comparing the design of both plate systems there are some differences to consider. The NCB-PT® is made out of titanium alloy, whereas the VA-LCP® is made out of steel. The smallest implant of the NCB-PT® has 5 holes with a length of 132 mm and is thus longer than the smallest VA-LCP® plate (4 holes/87 mm). The same applies to the longest implants where the NCB-PT® is considerably longer (292 mm) than the VA-LCP® (237 mm). In addition to the length, the NCB-PT® features a notable greater profile height [14] as well as considerable thicker cancellous and cortical screws (∅ 5.0 mm/4.0 mm) in comparison to the VA-LCP® (∅3.5 mm). Both plates allow the insertion of lag screws through the plate but with the VA-LCP® it is not possible to insert them through the proximal part of the plate.

The numerous differences between both implant systems result in different strengths and weaknesses for each plate. The decision to use one of the plate systems was consequently based upon the above-named characteristics of the plates. Furthermore, the surgeon’s choice was based on the individual assessment of the fracture and the patient, aiming to make most use of the strengths each plate system provides.

The mean operative time in this trial has been statistically significant longer in the NCB-PT® group. That is an unexpected result regarding the fact, that lag screws can be inserted directly through the plate into the metaphysis, which should save time in comparison to the VA-LCP®. In this context the higher number of complex type-C fractures in NCB-PT® patients (78% vs. 26%) (Table 1), going along with a more challenging reduction and additional posteromedial/−lateral osteosynthesis, could constitute a possible explanation. Since the double-plate fixation has proven to be the more biomechanically stable construct, particularly in the case of type-C /bicondylar fractures (Figs. 1 and 2) [29].

Furthermore, the insertion of the locking caps in the NCB-PT® system can be time-consuming.

Regarding the significant longer mean interval from injury to surgery in the NCB® patient group (5.8 d), the higher rate of type C-fractures with presumably worse soft tissue status should be considered. Partenheimer and coworkers reported of a mean injury to surgery interval of 7.5 days, including only type-C fractures in their study [6].

The use of significant longer implants in the NCB-PT® patients is most likely a result of the higher proportion of distal extended fractures (44% vs. 21%) (Table 2) and the higher amount of complex fractures in this group. The poor range of motion found in this study is not uncommon within these fractures. Papagelopoulos and coworkers explain this with the damaging of the extensor retinaculum and the joint surface by the initial trauma or by intraoperative exposure [30]. Only two scoring systems in this study showed statistically significant worse result for the NCB-PT® patients. Interpreting those results the heterogeneity of fracture severity, the use of significant longer implants in the NCB-PT® patient group as well as the different profile height should be taken into consideration. The complication rate of 18% in this study is comparable (Partenheimer et al. 22%, Jöckel et al. 25.6%) with similar studies of this fracture type [6, 27].

Regarding the radiologic results we could not find a different outcome between both groups.

The limitations of this study are the low number of patients, the uneven amount of the patients in each group, the heterogeneity of fracture types included and therefore an imbalance of additional posteromedial osteosynthesis. In the critical examination of the limitations and results of the study, the mismatch in the distribution of the fracture types between the two groups, especially the higher amount of complex type C-fractures in the NCB group, and its influence on the results of the study is particularly noticeable. Both Kraus et al. as well as Loibl et al. were able to determine significantly worse values of the Lysholm Score in severe fractures in their study [31, 32]. This bias should therefore be considered when interpreting the significant difference in the Lysholm Score between the two implants that we found in our study. On the other hand, data acquisition was complete and all clinical evaluations were performed by one person, excluding inter-observer bias.

## Conclusions

Summarizing, this is the first prospective clinical trial analysing the clinical and radiological outcome of different polyaxial locking plates in proximal tibia fractures. Reviewing the results of our study we found comparable results between both groups, in line with the current literature.

Further prospective randomized trials with higher patient numbers are needed to give a better picture and assessment regarding the clinical and radiological outcome of polyaxial locking plates in proximal tibia fractures.

## Data Availability

The datasets used and/or analysed during the current study are available from the corresponding author on reasonable request.

## Abbreviations

AP: Anterior-posterior
e.g.: exempli gratia
Fig.: Figure
MIS: Minimally invasive surgical
RoM: Range of motion
SEM: Standard error of the mean
